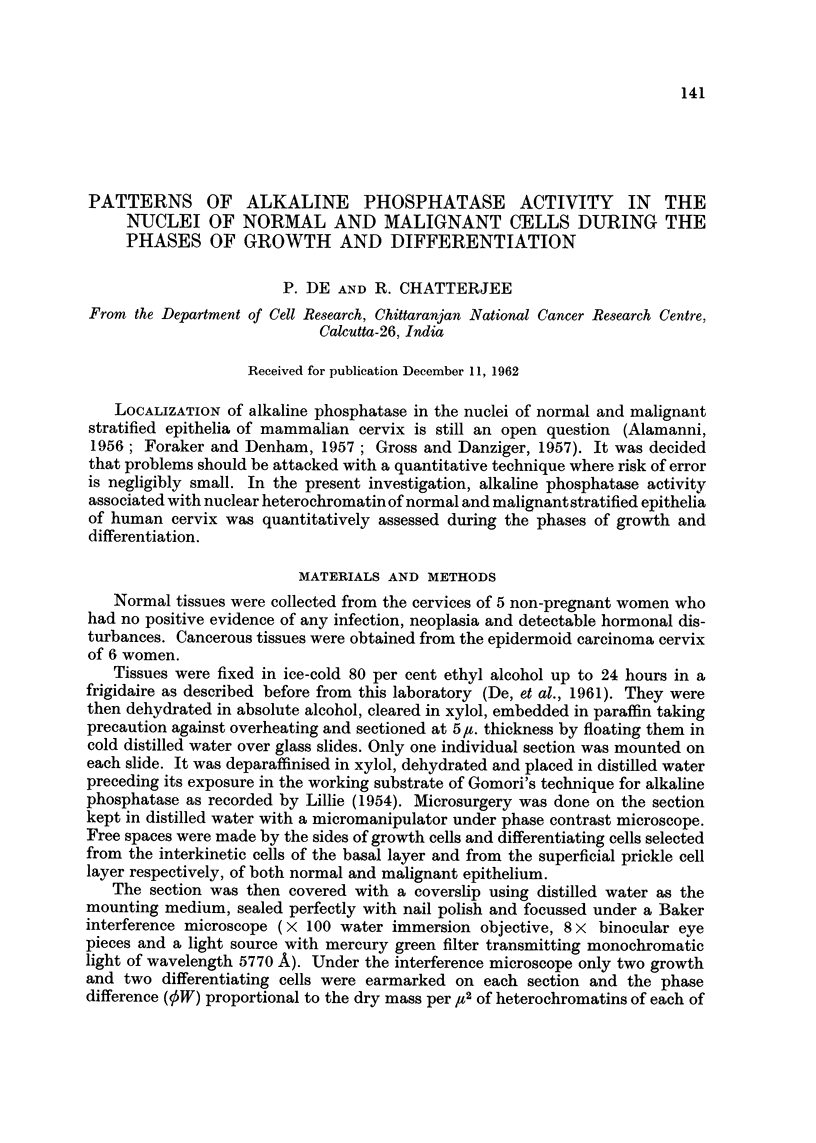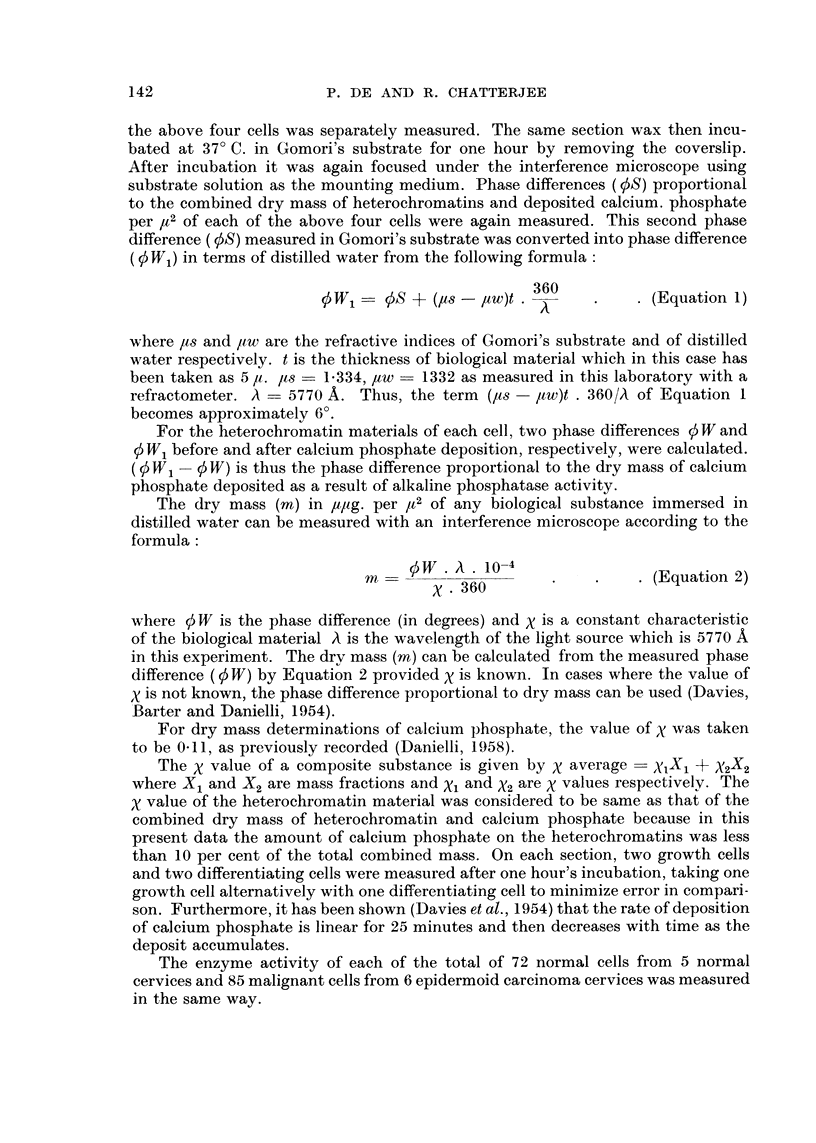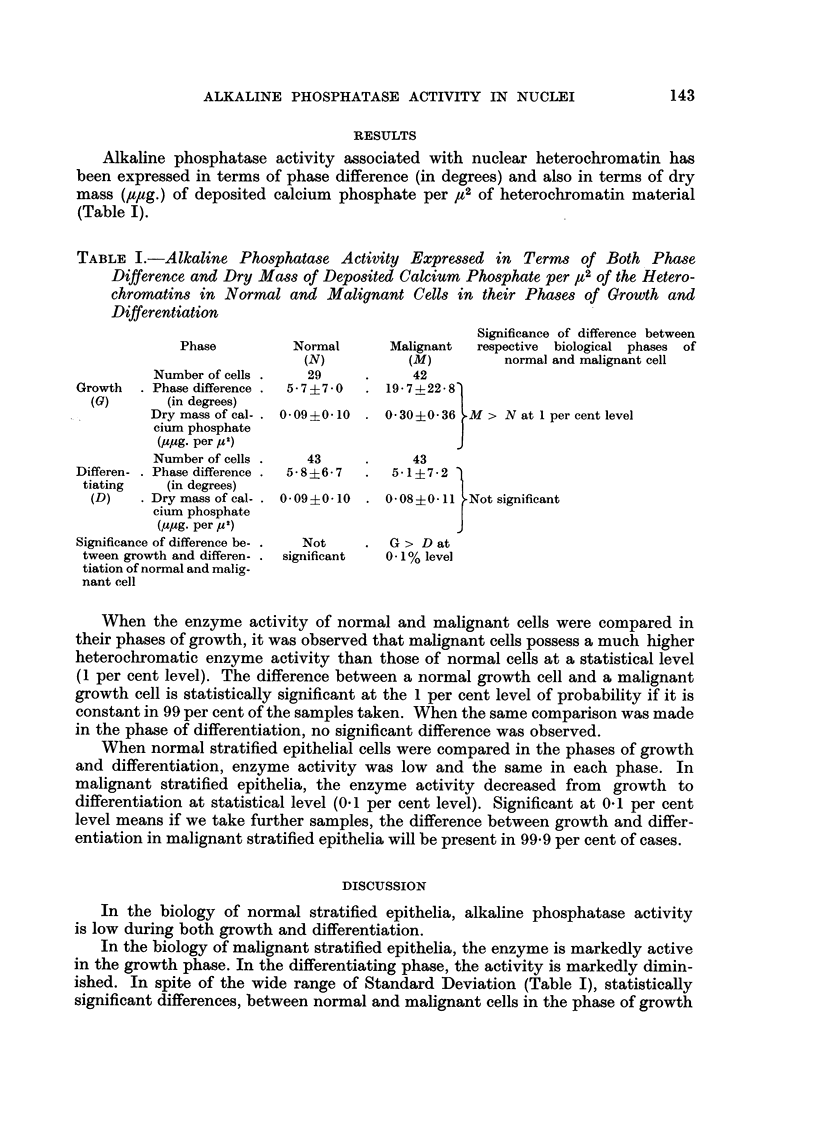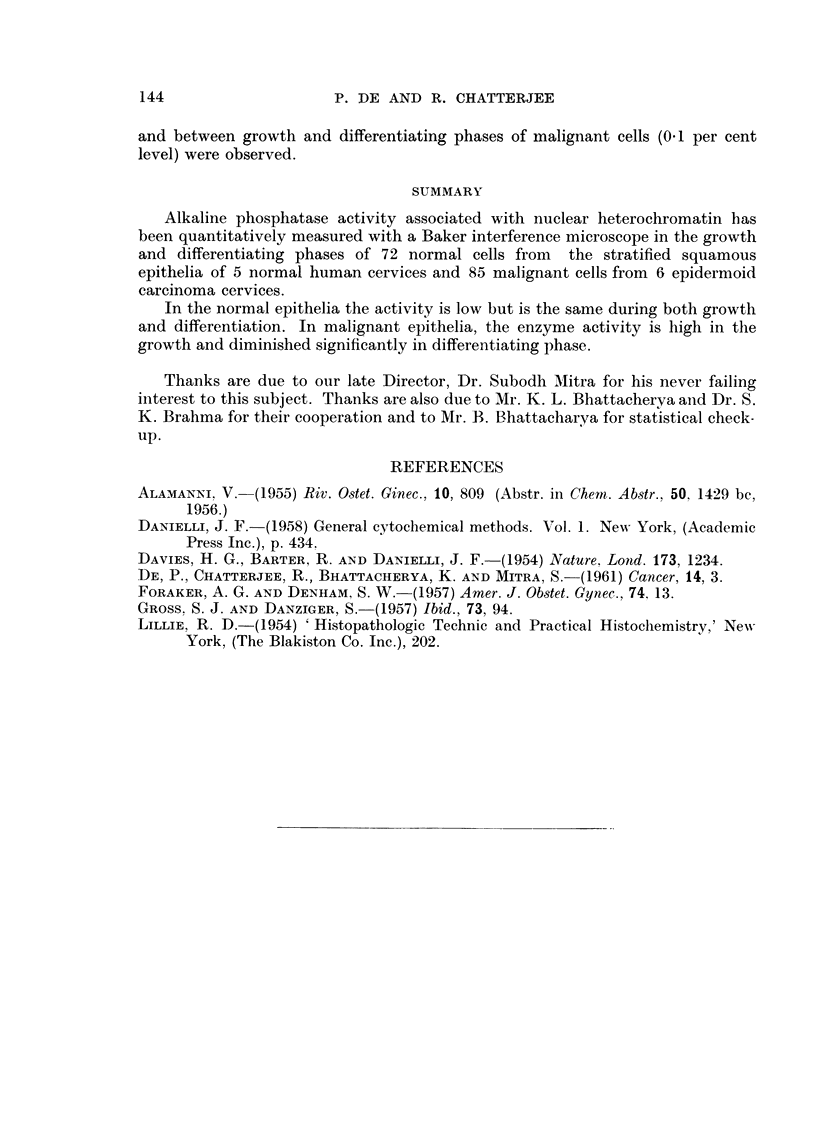# Patterns of Alkaline Phosphatase Activity in the Nuclei of Normal and Malignant Cells during the Phases of Growth and Differentiation

**DOI:** 10.1038/bjc.1962.14

**Published:** 1962-03

**Authors:** P. De, R. Chatterjee


### PATTERNS OF ALKALINE PHOSPHATASE ACTIVITY IN THE NUCLEI OF NORMAL AND MALIGNANT CELLS DURING THE PHASES OF GROWTH AND DIFFERENTIATION

P. DE AND R. CHATTERJEE

*From the Department of Cell Research, Chittaranjan National Cancer Research Centre, Calcutta-26, India*

Received for publication December 11, 1962

LOCALIZATION of alkaline phosphatase in the nuclei of normal and malignant stratified epithelia of mammalian cervix is still an open question (Alamanni, 1956 ; Foraker and Denham, 1957 ; Gross and Danziger, 1957). It was decided that problems should be attacked with a quantitative technique where risk of error is negligibly small. In the present investigation, alkaline phosphatase activity associated with nuclear heterochromatin of normal and malignant stratified epithelia of human cervix was quantitatively assessed during the phases of growth and differentiation.

### MATERIALS AND METHODS

Normal tissues were collected from the cervices of 5 non-pregnant women who had no positive evidence of any infection, neoplasia and detectable hormonal disturbances. Cancerous tissues were obtained from the epidermoid carcinoma cervix of 6 women.

Tissues were fixed in ice-cold 80 per cent ethyl alcohol up to 24 hours in a frigidaire as described before from this laboratory (De, *et al.*, 1961). They were then dehydrated in absolute alcohol, cleared in xylol, embedded in paraffin taking precaution against overheating and sectioned at $5\mu$. thickness by floating them in cold distilled water over glass slides. Only one individual section was mounted on each slide. It was deparaffinised in xylol, dehydrated and placed in distilled water preceding its exposure in the working substrate of Gomori's technique for alkaline phosphatase as recorded by Lillie (1954). Microsurgery was done on the section kept in distilled water with a micromanipulator under phase contrast microscope. Free spaces were made by the sides of growth cells and differentiating cells selected from the interkinetic cells of the basal layer and from the superficial prickle cell layer respectively, of both normal and malignant epithelium.

The section was then covered with a coverslip using distilled water as the mounting medium, sealed perfectly with nail polish and focussed under a Baker interference microscope ($\times$ 100 water immersion objective, 8$\times$ binocular eye pieces and a light source with mercury green filter transmitting monochromatic light of wavelength 5770 Å). Under the interference microscope only two growth and two differentiating cells were earmarked on each section and the phase difference ($\phi W$) proportional to the dry mass per $\mu^2$ of heterochromatins of each of the above four cells was separately measured. The same section wax then incubated at 37° C. in Gomori's substrate for one hour by removing the coverslip. After incubation it was again focused under the interference microscope using substrate solution as the mounting medium. Phase differences ($\phi S$) proportional to the combined dry mass of heterochromatins and deposited calcium. phosphate per $\mu^2$ of each of the above four cells were again measured. This second phase difference ($\phi S$) measured in Gomori's substrate was converted into phase difference ($\phi W_1$) in terms of distilled water from the following formula :

$$\phi W_1 = \phi S + (\mu s - \mu w)t \cdot \frac{360}{\lambda} \quad . \quad . \quad \text{(Equation 1)}$$

where $\mu s$ and $\mu w$ are the refractive indices of Gomori's substrate and of distilled water respectively. $t$ is the thickness of biological material which in this case has been taken as 5 $\mu$. $\mu s$ = 1·334, $\mu w$ = 1332 as measured in this laboratory with a refractometer. $\lambda$ = 5770 Å. Thus, the term $(\mu s - \mu w)t \cdot 360/\lambda$ of Equation 1 becomes approximately 6°.

For the heterochromatin materials of each cell, two phase differences $\phi W$ and $\phi W_1$ before and after calcium phosphate deposition, respectively, were calculated. ($\phi W_1 - \phi W$) is thus the phase difference proportional to the dry mass of calcium phosphate deposited as a result of alkaline phosphatase activity.

The dry mass ($m$) in $\mu\mu$g. per $\mu^2$ of any biological substance immersed in distilled water can be measured with an interference microscope according to the formula :

$$m = \frac{\phi W \cdot \lambda \cdot 10^{-4}}{\chi \cdot 360} \quad . \quad . \quad . \quad \text{(Equation 2)}$$

where $\phi W$ is the phase difference (in degrees) and $\chi$ is a constant characteristic of the biological material $\lambda$ is the wavelength of the light source which is 5770 Å in this experiment. The dry mass ($m$) can be calculated from the measured phase difference ($\phi W$) by Equation 2 provided $\chi$ is known. In cases where the value of $\chi$ is not known, the phase difference proportional to dry mass can be used (Davies, Barter and Danielli, 1954).

For dry mass determinations of calcium phosphate, the value of $\chi$ was taken to be 0·11, as previously recorded (Danielli, 1958).

The $\chi$ value of a composite substance is given by $\chi$ average = $\chi_1 X_1 + \chi_2 X_2$ where $X_1$ and $X_2$ are mass fractions and $\chi_1$ and $\chi_2$ are $\chi$ values respectively. The $\chi$ value of the heterochromatin material was considered to be same as that of the combined dry mass of heterochromatin and calcium phosphate because in this present data the amount of calcium phosphate on the heterochromatins was less than 10 per cent of the total combined mass. On each section, two growth cells and two differentiating cells were measured after one hour's incubation, taking one growth cell alternatively with one differentiating cell to minimize error in comparison. Furthermore, it has been shown (Davies *et al.*, 1954) that the rate of deposition of calcium phosphate is linear for 25 minutes and then decreases with time as the deposit accumulates.

The enzyme activity of each of the total of 72 normal cells from 5 normal cervices and 85 malignant cells from 6 epidermoid carcinoma cervices was measured in the same way.

### RESULTS

Alkaline phosphatase activity associated with nuclear heterochromatin has been expressed in terms of phase difference (in degrees) and also in terms of dry mass ($\mu\mu$g.) of deposited calcium phosphate per $\mu^2$ of heterochromatin material (Table I).

TABLE I.—*Alkaline Phosphatase Activity Expressed in Terms of Both Phase Difference and Dry Mass of Deposited Calcium Phosphate per $\mu^2$ of the Heterochromatins in Normal and Malignant Cells in their Phases of Growth and Differentiation*

| | Phase | Normal ($N$) | Malignant ($M$) | Significance of difference between respective biological phases of normal and malignant cell |
|---|---|---|---|---|
| Growth ($G$) | Number of cells | 29 | 42 | |
| | Phase difference (in degrees) | 5·7±7·0 | 19·7±22·8 | $M > N$ at 1 per cent level |
| | Dry mass of calcium phosphate ($\mu\mu$g. per $\mu^2$) | 0·09±0·10 | 0·30±0·36 | |
| Differentiating ($D$) | Number of cells | 43 | 43 | |
| | Phase difference (in degrees) | 5·8±6·7 | 5·1±7·2 | Not significant |
| | Dry mass of calcium phosphate ($\mu\mu$g. per $\mu^2$) | 0·09±0·10 | 0·08±0·11 | |
| Significance of difference between growth and differentiation of normal and malignant cell | | Not significant | G > $D$ at 0·1% level | |

When the enzyme activity of normal and malignant cells were compared in their phases of growth, it was observed that malignant cells possess a much higher heterochromatic enzyme activity than those of normal cells at a statistical level (1 per cent level). The difference between a normal growth cell and a malignant growth cell is statistically significant at the 1 per cent level of probability if it is constant in 99 per cent of the samples taken. When the same comparison was made in the phase of differentiation, no significant difference was observed.

When normal stratified epithelial cells were compared in the phases of growth and differentiation, enzyme activity was low and the same in each phase. In malignant stratified epithelia, the enzyme activity decreased from growth to differentiation at statistical level (0·1 per cent level). Significant at 0·1 per cent level means if we take further samples, the difference between growth and differentiation in malignant stratified epithelia will be present in 99·9 per cent of cases.

### DISCUSSION

In the biology of normal stratified epithelia, alkaline phosphatase activity is low during both growth and differentiation.

In the biology of malignant stratified epithelia, the enzyme is markedly active in the growth phase. In the differentiating phase, the activity is markedly diminished. In spite of the wide range of Standard Deviation (Table I), statistically significant differences, between normal and malignant cells in the phase of growth and between growth and differentiating phases of malignant cells (0·1 per cent level) were observed.

SUMMARY

Alkaline phosphatase activity associated with nuclear heterochromatin has been quantitatively measured with a Baker interference microscope in the growth and differentiating phases of 72 normal cells from the stratified squamous epithelia of 5 normal human cervices and 85 malignant cells from 6 epidermoid carcinoma cervices.

In the normal epithelia the activity is low but is the same during both growth and differentiation. In malignant epithelia, the enzyme activity is high in the growth and diminished significantly in differentiating phase.

Thanks are due to our late Director, Dr. Subodh Mitra for his never failing interest to this subject. Thanks are also due to Mr. K. L. Bhattacherya and Dr. S. K. Brahma for their cooperation and to Mr. B. Bhattacharya for statistical check-up.

### REFERENCES

ALAMANNI, V.—(1955) *Riv. Ostet. Ginec.*, **10**, 809 (Abstr. in *Chem. Abstr.*, **50**, 1429 bc, 1956.)
DANIELLI, J. F.—(1958) General cytochemical methods. Vol. 1. New York, (Academic Press Inc.), p. 434.
DAVIES, H. G., BARTER, R. AND DANIELLI, J. F.—(1954) *Nature, Lond.* **173**, 1234.
DE, P., CHATTERJEE, R., BHATTACHERYA, K. AND MITRA, S.—(1961) *Cancer*, **14**, 3.
FORAKER, A. G. AND DENHAM, S. W.—(1957) *Amer. J. Obstet. Gynec.*, **74**, 13.
GROSS, S. J. AND DANZIGER, S.—(1957) *Ibid.*, **73**, 94.
LILLIE, R. D.—(1954) 'Histopathologic Technic and Practical Histochemistry,' New York, (The Blakiston Co. Inc.), 202.